# A three-stage DEA-based efficiency evaluation of social security expenditure in China

**DOI:** 10.1371/journal.pone.0226046

**Published:** 2020-02-11

**Authors:** Yangming Hu, Yingjun Wu, Wei Zhou, Tao Li, Liqing Li

**Affiliations:** College of Public Management & Law, Hunan Agricultural University, Changsha, Hunan, China; Wuhan University, CHINA

## Abstract

There is an increasingly growth of China’s social security expenditure(SSE) during the past decade. Regarding to the great responsibility and impact on citizens’ welfare and economic development, the efficiency of social security expenditure has inevitably become the focus of growing attention. Based on Chinese provincial panel data over the period 2007–2016, a three-stage DEA model was conducted and found that the efficiency level of 29 provinces/municipalities did not reach the efficiency frontier. Environmental factors and statistical noises have a significant impact on the efficiency of SSE, if environmental factors and statistical noises are not considered, the efficiency of SSE in China is likely to be underestimated. The regional differences in the efficiency of SSE were significant and ranked by descending order as follows: central region, eastern region and western region.

## Introduction and literature review

There is an increasingly growth of China’s social security expenditure during the past decade. Although the sources of social security funds are diversified, government funding remains the most important source. According to regional fiscal expenditure data published in the China Statistical Yearbook, items related to social security expenditure (SSE) include social security and employment expenditure and health expenditure. As the main supplier of public goods in the region, local governments are responsible for more than 90% of SSE. With an increase in investment in social security, the efficiency of social security expenditure has inevitably become the focus of growing attention.

Prior researchers have studied the influence that social security expenditure plays on national economic growth[1–5]. Bellettini and Ceroni(2000) adopted a sampleset from 61 countries from 1970 to 1985 and 20 OECD(Organization for Economic Cooperation and Development) countries over the period 1960–1990, they found a postive sign between security expenditure and economic growth[2]. Zhang et al.(2019) used dynamic panel data from 2007 to 2016 and found social security is favorable for sustained economic growth of China[6]. Chinese scholars have also put attention to the utility of social security system in long-term economy growth, pension reform and fertility policy[7–9].

As the fact that social security is legislated in every country insinuates that the social security system is the responsibility of the state. In addition to the establishment, implementation, and supervision of the system, such a responsibility is also reflected in the fiscal expenditure of the government. Thus appropriate evaluation of the efficiency of SSE is of practical significance for optimizing the social security structure and improving the management of corresponding funding inputs.

The criteria of effective social security funds is the ability of balancing well-matched benefits with the protection of distorting private saving[10]. Shiller(2005) evaluated the returns of US social security account by undertaking a simulation based on stock market, bond market and money market data from 1871 to 2004[11]. Dixon(2011) comparative studied 27 asian countries’ security system and found a perennial problem of inadequate public financing and implementing[12]. Ginneken(2003) studied the importance of social security programmes for developing countries and pointed out that national and international policies were needed to enhance its cost-effectiveness[13].

As a method of analysis, the Data Envelopment Analysis (DEA) model has been widely used to efficiency analysis since its conception by Charnes et al. in 1978 [14–16]. Moreover, the method of of Stochastic Frontier Analysis (SFA), which is a model using frontier concept in a regression framwork, was introduced to estimate efficiency[17–18]. Scholars have compared DEA and SFA methods in evaluating efficiency[19–20]. Jacobs(2000) applied data envelopment analysis and stochastic frontier analysis to measure different aspects of efficiency of NHS hospital efficiency in UK[21].

In recent years, a number of Chinese scholars have adopted the traditional DEA and two-stage DEA models to study the efficiency of SSE at both regional and national levels—from an input-output perspective [22–27]. However, the application of the traditional DEA and two-stage DEA models for measuring the efficiency of SSE and the factors that influence SSE in China have certain limitations. Traditional DEA model can only measure the efficiency, but cannot measure the factors that affect the efficiency. Two-stage DEA model can analyze the factors affecting efficiency, but it cannot exclude the influence of environmental factors and statistical noises on the efficiency value. These limitations tend to lead to overestimation/underestimation of the actual efficiency, and both methods thus require further improvement. Zheng et al.(2018) pointed out a four stage DEA model can effectively eliminate the impact of environmental factors.[28]

This study employed a three-stage DEA model, which was first proposed by Fried et al.(2002)[29], to incorporate environmental effects and statistical noise into efficiency evaluation. A sampleset of 31 provinces and municipalities in China from 2007 to 2016 were analyzed to calculate the efficiency of SSE in China throughout the study period.

## Methodology

According to Fried et al.(2002), a conventional oriented DEA analysis was conducted using input quantity data and output quantity data only in the first stage analysis while TE (Technical Efficiency) can be decomposed into PTE (Pure Technical Efficiency) and Scale Efficiency (SE), that is, TE = PTE×SE.

The objective of second stage analysis was to decompose Stage 1 slacks (environmental influences, managerial inefficiencies, and statistical noises) which were arised from measurement errors in the input and output data. Following Fried et al., we built up SFA regression formulation as:

$$s_{ik}=f_{i}\left(z_{k};\;\beta_{i}\right)+v_{ik}+\mu_{ik}$$

Where, *s*_*ik*_ represents the slack variable of input item i of the k^th^ decision unit(i = 1, 2, …, m; K = 1, 2, …, n); *z*_*k*_ = (*z*_1*k*_, *z*_2*k*_, …, *z*_*pk*_) represents observable environmental variables in the amount of P; *f*_*i*_(*z*_*k*_; *β*_*i*_) represents the effect of environment variable on input slack variable *s*_*ik*_, usually make *f*_*i*_(*z*_*k*_; *β*_*i*_) = *z*_*k*_*β*_*i*_. *v*_*ik*_ + *μ*_*ik*_ represents composed error, *v*_*ik*_ represents statistical noise, and $v_{ik}\sim N(0,\sigma_{vi}^{2})$; *μ*_*ik*_ represents managerial inefficiency. Assuming that it follows a truncated normal distribution as μik~N+(μi,σui2), and *v*_*ik*_, *μ*_*ik*_ are distributed independently of each other. Let ${\gamma =\sigma }_{ui}^{2}/(\sigma_{ui}^{2}+\sigma_{vi}^{2})$, the closer the value of *γ* is to 1, the more managerial factors dominate the error part of the model; the closer the value of *γ* is to 0, the more statistical noise dominates the error part of the model.

In the case that the maximum likelihood estimation method is used to calculate the parameters *β*_*i*_, *σ*^2^ and *γ*, it is necessary to calculate the estimated value of statistical noise *v*_*ik*_ and managerial inefficiency *μ*_*ik*_ for effective adjustment of input slacks.

Fried et al.(2002) adopted Jondrow et al.(1982)[30] approach to decompose composed error structure *v*_*ik*_ + *μ*_*ik*_.

$$E\left[v_{ik}|v_{ik}+\mu_{ik}\right]=s_{ik}-f_{i}\left(z_{k};\beta_{i}\right)-\text{E}\left[\mu_{ik}|v_{ik}+\mu_{ik}\right]$$

However, Fried et al. provided no solution of calculating *E*[*μ*_*ik*_ | *v*_*ik*_ + *μ*_*ik*_]. Current literatures have usually applied Jondrow et al.(1982) formula for managerial inefficiency, which was based on the assumption of *ε*_*i*_ = *v*_*i*_ − *μ*_*i*_ (*ε*_*i*_ *for composed error*), while Fried et al. was *ε*_*i*_ = *v*_*i*_ + *μ*_*i*_.

This study applied Dengyue L.(2012)[31] approach as following:

$$E[\mu_{ik}|v_{ik}+\mu_{ik}]=\frac{\sigma \lambda }{1+\lambda^{2}}\left[\frac{\varphi (\frac{\varepsilon_{k}\lambda }{\sigma })}{\emptyset (\frac{\varepsilon_{k}\lambda }{\sigma })}+\frac{\varepsilon_{k}\lambda }{\sigma }\right]$$

Where, $\lambda =\frac{\sigma_{u}}{\sigma_{v}}$, *ε*_*k*_ = *v*_*ik*_ + *μ*_*ik*_, $\sigma^{2}=\sigma_{u}^{2}+\sigma_{v}^{2}$, *φ*, ∅ are the density function and distribution function of the standard normal distribution respectively.

The adjusted input value is obtained through the following formula:

$$x_{ik}^{*}=x_{ik}+[max(z_{k}\beta_{i})-z_{k}\beta_{i}]+[max(v_{ik})-v_{ik}]$$

When, $x_{ik}^{*}$ is the input after adjusting the original input value *x*_*ik*_ in the second stage. [*max*(*z*_*k*_*β*_*i*_) − *z*_*k*_*β*_*i*_] represents to adjust all decision making units to the same external environment. [*max*(*v*_*ik*_) − *v*_*ik*_] represents to adjust all statistical noise of decision making units to the same situation.

In the third stage, we replaced the original input *x*_*ik*_ in the first stage with the adjusted $x_{ik}^{*}$ obtained in the second stage, then repeated the first stage analysis by applying DEA to the adjusted data. This phase improved measures of managerial efficiency while both environmental effects and statistical noise had been purged in the second stage SFA regression.

## Indicator selection and data source

### Input and output indicators

Selecting appropriate indicators is crucial for achieving a comprehensive and objective evaluation of the efficiency of SSE. The input indicator, social security, is the sum of SSE, including social security and employment expenditure and health expenditure. To eliminate the influence of regional population size, this study selected per capita SSE as an input variable. Social insurance, social assistance, and social welfare are the main components of SSE. Moreover, since the level of consumption is a direct reflection of the efficiency of SSE, this study utilized several factors as the output variables to measure the efficiency of SSE, namely, the coverage of endowment insurance, number of hospital beds per 1,000 people, coverage of minimum living allowance, employment rate, level of consumption, and coverage gap between urban and rural areas. The description and calculation of each indicator are shown in Table 1.

**Table 1:** Input and output indicators and environment variables.

Category	Indicator	Unit	Calculation
Input Indicator	Per Capita SSE	Yuan	Expenditure on social security, employment, and healthcare/total population in the region
Output Indicator	Coverage of Endowment Insurance	%	Population covered by endowment insurance/population over 15 years old
	Hospital Beds per 1,000 People	Piece	-
	Coverage of Minimum Living Allowance	%	Population covered by minimum living allowance/total population in the region
	Employment Rate	%	1—unemployment rate
	Level of Consumption	Yuan	-
	Gap between Urban and Rural Areas	%	Per capita net income of rural residents/per capita disposable income of urban residents
Environmental Variables	Per Capita GDP	Yuan	Regional GDP/total population of the region
	Urbanization Level	%	Urban population/total population of the region
	Marketization Level	%	Added value of tertiary industries/regional GDP
	Financial Autonomy	%	Fiscal revenue/fiscal expenditure

The DEA model requires that the input and output variables are positively correlated—that is, an increase in the input variables cannot cause a decrease in the output variables. The study used SPSS 22.0 to perform a Pearson correlation test on the input and output variables. The results are shown in Table 2. It can be seen that the correlation coefficients between the input and output variables are positive and statistically significant (*p* < 0.01, 2-tailed), indicating that the selected variables are appropriate.

**Table 2:** Pearson correlation test of the input and output indicators.

Item	Coverage of Endowment Insurance	Hospital Beds per 1,000 People	Minimum Living Allowance Coverage	Employment Rate	Level of Consumption	Difference between Urban and Rural Areas
Per Capita SSE	0.560** (0.000)	0.575** (0.000)	0.062 (0.274)	0.329** (0.000)	0.523** (0.000)	0.244** (0.000)

Note:

**: *p* < 0.01

### Environment variables

In addition to being affected by internal factors, such as budgets and expenditure structures, the available social security funds are also influenced by external environmental factors. External factors (environmental variables) refer to the factors that are neither input nor output factors but affect the efficiency of SSE and are not controllable by the corresponding government body. The majority of Chinese scholars have tended to approach environmental variables from an economic or social perspective. In this study, the following environmental variables were considered:

1. Economic Development Level: It is generally believed that regions with a stronger economy have more basic conditions that are favorable to the improvement of SSE efficiency. However, since regions with a stronger economy are more likely to invest more into social security, the risk of over-investment and waste also increases, leading to a reduction in the efficiency of SSE. In this study, the per capita GDP of the region was employed to characterize regional economic development.
2. Urbanization Level: The urbanization process is usually accompanied by the accumulation of capital and labor. This aggregated effect is likely to promote employment and economic development in the region, as well as the improvement of public facilities and services. Therefore, a higher level of urbanization signifies an increased likelihood of the implementation of social security policies.
3. Marketization Level: The level of marketization in a region reflects the comprehensiveness of the legal environment and maturity of the factor market, which can affect the government’s fiscal efficiency. In addition, marketization promotes the demand for labor and thus facilitates social insurance coverage. Generally, a higher level of marketization signifies a higher efficiency of capital allocation and fiscal expenditure.
4. Financial Autonomy: Since the reform of the tax system, the Chinese government has centralized financial power and decentralized administrative power. For this reason, although regional governments have less financial autonomy, their social development related responsibilities has remain unchanged. Such an imbalance between financial and administrative power is likely to lead to behavioral preferences for local governments and to exacerbate competition among local governments, thereby affecting the efficiency of SSE. For this reason, this study introduced financial autonomy as a variable to measure fiscal decentralization.

### Data source

The method used by the Chinese government to calculate SSE was changed in 2007. Specifically, categories such as pensions and relief funds for social welfare, social security subsidiary expenses, and pensions for administrative and public institutions were merged into one category (social security and employment expenditure). The change has engendered large discrepancies between the data before and after 2007. To ensure data consistency, this study selected SSE data on 31 provinces and municipalities from 2007 to 2016. All data were extracted from the China Statistical Yearbook and China Health and Family Planning Yearbook for the corresponding year. To further measure the differences in the efficiency of SSE among regions, the 31 provinces and municipalities were divided into eastern, central, and western regions (Table 3).

**Table 3:** Categorization of the 31 provincial administrative regions in China.

Region	Number	Provinces and Municipalities
Eastern	11	Beijing, Tianjin, Hebei, Liaoning, Shanghai, Jiangsu, Zhejiang, Fujian, Shandong, Guangdong, Hainan
Central	8	Shanxi, Jilin, Heilongjiang, Anhui, Jiangxi, Henan, Hubei, Hunan
Western	12	Inner Mongolia, Guangxi, Chongqing, Sichuan, Guizhou, Yunnan, Tibet, Shaanxi, Gansu, Qinghai, Ningxia, Xinjiang

## Empirical study of the efficiency of China’s social security expenditure

### Analysis results of the DEA model: Stage 1

The DEAP 2.1 software package was employed to measure the efficiency of SSE and returns to scale for the 31 provinces and municipalities. Table 2 shows the mean values of the efficiency of SSE during the observation period and the returns to scale in 2016. The mean value for technical efficiency (TE), pure technical efficiency (PTE), and scale efficiency (SE) for Stage 1, without considering the environmental variables and statistical noises, was 0.774, 0.881, and 0.885, respectively. PTE and SE were the factors that limited the efficiency of SSE in China. In addition, Zhejiang, Shandong and Guizhou were at the frontier of efficiency. Six provinces and municipalities, such as Beijing, Shanghai, and Jiangsu, were high in PTE, indicating they had weak DEA efficiency. PTE and SE of the remaining provinces and municipalities could be further improved. From a regional perspective, the mean TE for the central region was the highest (0.837), followed by the eastern (0.798) and western (0.711) regions.

### Analysis results of the SFA model: Stage 2

At stage 2, the slack variable (per capita SSE) obtained from stage 1 was introduced as the dependent variable, and per capita GDP, urbanization level, marketization level, and financial autonomy were introduced as independent variables in the model. To ensure the accuracy of the calculation, a yearly cross-sectional regression technique was adopted. The software application, Frontier 4.1, was utilized to perform the stochastic frontier analysis (SFA). Owing to word count limitations, only the results for 2016 are presented in Table 4.

**Table 4:** Results of the SFA model: Stage 2.

Variables	Per Capita SSE	
	Estimated Coefficient	Standard Error
Constant	2426.349***	1.006
Per Capita GDP	0.054***	0.000
Urbanization Level	-111.790***	5.935
Marketization Level	30.602***	3.921
Financial Autonomy	-19.457***	2.821
*σ*^2^	1265776.500***	1
*γ*	0.999***	0.000
Log Likelihood	-241.490	
One-sided LR Test	13.266***	

Note:

*: *p* < 0.1,

**: *p* < 0.05,

***: *p* < 0.01.

According to Table 4, the LR unilateral generalized likelihood ratio test of the SFA model passed the significance test at the 1% level, and rejected the null hypothesis that there was no managerial inefficiency, indicating that it was reasonable to apply the SFA model in the second stage. Both *σ*^2^ and *γ* value passed the significance test (*γ* = 0.999), indicating that compared with random error, managerial inefficiency in the mixed error term has a dominant influence on the slack variable. In addition, the estimated coefficients of the four environmental variables also passed the significance test, indicating that environmental factors have a significant impact on the slack values of social security and employment expenditure; therefore, applying the SFA model to separate the environmental variables and statistical noises is reasonable. Since environmental variables are regressions in the input slack value; if the estimated coefficient of an environmental variable is negative, then increasing the environmental variable can reduce the input slack, which is beneficial for improving efficiency. If the estimated coefficient is positive, then increasing the environmental variable will increase the input redundancy, which is not conducive to improving efficiency. The findings, based on Table 4, are as follows:

1. Per Capita GDP: The regression coefficients of regional per capita GDP and input slack are positive and statistically significant, indicating that an increase in the economic level tends to lead to a significant increase in the redundancy of social security inputs, which negatively affects the improvement of efficiency.
2. Urbanization Level: The regression coefficient of urbanization level is negative (*p* < 0.01), suggesting that the urbanization level has a positive effect on the efficiency of SSE. The agglomeration effect of the urbanization process has promoted the improvement of public goods and services. During this process, rural residents’ mentality has shifted to becoming more closely correlated with the mentality of urban citizens; therefore, rural residents are driven toward narrowing the gap in living conditions with urban citizens, which is conducive to improving the efficiency of SSE.
3. Marketization Level: The estimated coefficient of marketization level is positive and statistically significant, indicating that the marketization level significantly facilitates input slack. This finding suggests that excessive development of the tertiary industry does not necessarily lead to an increase in the efficiency of SSE.
4. Financial Autonomy: The estimated coefficient of financial autonomy is negative and statistically significant, suggesting that financial autonomy significantly promotes the improvement of efficiency. When local governments have more financial autonomy and a larger team size, their SSE efficiency increases.

### Analysis results of the DEA model: Stage 3

The SFA model in stage 2 eliminated the influence of environmental factors and statistical noises on efficiency. The adjusted input value was then introduced into the model to replace the original input value at stage 1. The efficiency of SSE by province and municipality was then obtained (Table 5).

**Table 5:** DEA efficiency of the 31 provinces/municipalities prior to and following the adjustment.

Regions	Stage 1				Stage 3			
	TE	PTE	SE	RTS	TE	PTE	SE	RTS
Beijing	0.509	1.000	0.509	drs	0.639	1.000	0.639	drs
Tianjing	0.601	0.931	0.648	drs	0.741	0.969	0.763	drs
Hebei	0.951	0.957	0.993	-	0.935	0.940	0.994	irs
Liaoning	0.567	0.938	0.612	drs	0.641	0.940	0.690	drs
Shanghai	0.635	1.000	0.635	drs	0.778	1.000	0.778	drs
Jiangsu	0.988	1.000	0.988	-	0.985	1.000	0.985	-
Zhejiang	1.000	1.000	1.000	-	0.999	1.000	0.999	drs
Fujian	0.960	0.962	0.998	-	0.917	0.919	0.998	irs
Shandong	1.000	1.000	1.000	-	1.000	1.000	1.000	-
Guangdong	0.990	1.000	0.990	drs	0.909	0.995	0.914	drs
Hainan	0.580	0.783	0.777	drs	0.624	0.807	0.803	drs
**Mean of Eastern area**	**0.798**	**0.961**	**0.832**		**0.834**	**0.961**	**0.869**	
Shanxi	0.745	0.787	0.948	irs	0.742	0.767	0.967	irs
Jilin	0.703	0.909	0.783	drs	0.798	0.941	0.852	drs
Heilongjiang	0.779	0.975	0.804	drs	0.820	0.975	0.845	drs
Anhui	0.854	0.882	0.971	drs	0.826	0.851	0.975	irs
Jiangxi	0.932	0.978	0.954	drs	0.894	0.951	0.942	drs
Henan	0.979	0.986	0.994	-	0.990	0.994	0.996	-
Hubei	0.813	0.911	0.898	drs	0.847	0.922	0.922	drs
Hunan	0.891	0.911	0.979	-	0.904	0.915	0.988	-
**Mean of Middle area**	**0.837**	**0.917**	**0.916**		**0.852**	**0.914**	**0.936**	
Inner Mongolia	0.525	0.573	0.917	irs	0.667	0.700	0.951	irs
Guangxi	0.966	0.971	0.996	drs	0.957	0.962	0.995	drs
Chongqi	0.653	0.745	0.900	drs	0.695	0.767	0.924	drs
Sichuan	0.832	0.883	0.946	drs	0.882	0.904	0.977	drs
Guizhou	1.000	1.000	1.000	-	1.000	1.000	1.000	-
Yunnan	0.804	0.816	0.985	drs	0.839	0.842	0.996	irs
Tibet	0.368	0.428	0.873	drs	0.514	0.566	0.907	drs
Shanxi	0.679	0.709	0.959	drs	0.750	0.760	0.987	-
Gansu	0.849	1.000	0.849	-	0.927	1.000	0.927	-
Qinghai	0.362	0.619	0.704	drs	0.462	0.665	0.781	drs
Ningxia	0.621	0.653	0.954	drs	0.714	0.732	0.977	irs
Sinkiang	0.869	1.000	0.869	drs	0.980	1.000	0.980	drs
**Mean of Western area**	**0.711**	**0.783**	**0.913**		**0.782**	**0.825**	**0.950**	
**Mean of Nationwide**	**0.774**	**0.881**	**0.885**		**0.818**	**0.896**	**0.918**	

Note: “TE,” “PTE,” “SE,” and “RTS” represent technical efficiency, pure technical efficiency, scale efficiency, and returns to scale, respectively. In addition, “irs,” “drs,” and “-” signify that the returns to scale increased, decreased, or remained unchanged, respectively.

#### Analysis of overall efficiency

1. Analysis of Technical Efficiency: TE was used to measure the overall SSE of the provinces/municipalities in terms of the investment, use, and management of capital. It can be seen that the mean value of TE for most provinces improved by a certain degree. The overall TE increased from 0.774 at stage 1 to 0.818 at stage 3. On the provincial level, the number of provinces/municipalities at the efficiency frontier decreased from three at stage 1 to two at stage 3 (Zhejiang was removed from the list). The finding confirms that the SSE efficiency of Shandong and Guizhou remained consistently high, as they remained at the efficient frontier. The efficiency of Tibet, Tianjin, Shanghai, Inner Mongolia, Beijing, and Xinjiang significantly changed after the model adjustments, indicating that environmental factors have a considerable impact on these provinces/municipalities. In addition, the TE of Hebei, Fujian, Guangdong, Anhui, Jiangxi, and Guangxi decreased after the model adjustment. The decline in efficiency was most prominent in Guangdong (from 0.990 to 0.909).
2. Analysis of Pure Technical Efficiency: PTE reflected the allocation and management of SSE for each province/municipality. The overall PTE increased from 0.881 at stage 1 to 0.896 at stage 2. Specifically, the PTE of Tibet, Ningxia, and Inner Mongolia notably increased; as a result, the overall efficiency of the three provinces at stage 3 was greatly improved. In addition, Tibet showed the most prominent improvement (from 0.428 at stage 1 to 0.566 at stage 3). However, the PTE of Hebei, Fujian, Anhui, Shanxi, and Guangxi decreased, and the decline in efficiency of Fujian was most notable.
3. Analysis of Scale Efficiency: SE reflects the gap between the current scale and optimal scale of SSE in each province/municipality. The overall SE increased from 0.885 at stage 1 to 0.918 at stage 3. Only the SE of Guangdong and Jiangxi decreased during stage 3. In addition, the decrease in the efficiency of Guangdong was most significant (from 0.990 at stage 1 to 0.914 at stage 3). Among the provinces/municipalities with the greatest increase in SE, Shanghai showed the most prominent improvement (from 0.635 at stage 1 to 0.778 at stage 3).

#### Analysis of regional differences

Before the adjustment, the means of TE for the regions showed the following pattern(ranked by descending order): central region, eastern region and western region. Moreover, the TE of the western region was lower than the national average; the SE of the eastern region and the PTE of the western region were also lower than the national average. It can be seen that SE and PTE were the key factors that restricted the efficiency of SSE in the eastern and western regions, respectively. After eliminating the influences of environmental factors and statistical noises, the efficiency of the three regions increased. Specifically, the increase in TE for the western region was most apparent; however, the efficiency value remained low. The efficiency of the central region remained greater than that of the eastern region, and that of the western region was the lowest.

To further analyze the efficiency of each region, this study set 0.9 as the relative threshold based on the mean value of PTE and SE for the regions (above 0.9 was high efficiency and below 0.9 was low efficiency) and categorized the efficiency level of the provinces/municipalities into four groups: high PTE and SE, high PTE and low SE, low PTE and high SE, and low PTE and SE (Table 6). For the high PTE and SE group, the PTE and SE of the provinces and municipalities in this category are both greater than 0.9, indicating that the management and input scale of SSE in these provinces/municipalities is more than satisfactory. Fifteen provinces/municipalities, such as Hebei, Jiangsu, and Zhejiang, are included this category. For the high PTE and low SE group, the provinces and municipalities in this category have a PTE greater than 0.9 and an SE smaller than 0.9, indicating that their allocation and management of SSE is relatively good. Six provinces/municipalities are included in this category, including Beijing and Tianjin. For the low PTE and high SE group. the provinces and municipalities in this category have a PTE smaller than 0.9 and an SE greater than 0.9. Eight provinces and municipalities belong to this category, including Shanxi, Inner Mongolia, and Anhui. For the low PTE and SE group, the PTE and SE of the provinces and municipalities in this category are both smaller than 0.9, indicating that the provinces needed to improve both their PTE and their SE. Only Hainan and Qinghai belong to this category.

**Table 6:** Efficiency level distribution of each area.

**FHigh SE**	**Low PTE, High SE (8)**: Shanxi, Inner Mongolia, Anhui, Chongqing, Yunnan, Tibet, Shaanxi, and Ningxia	**High PTE, High SE (15)**: Hebei, Jiangsu, Zhejiang, Fujian, Jiangxi, Shandong, Henan, Hubei, Hunan, Guangdong, Guangxi, Sichuan, Guizhou, Gansu, and Xinjiang
**Low SE**	**Low PTE, Low SE (2)**: Hainan and Qinghai	**High PTE, Low SE (6)**: Beijing, Tianjin, Liaoning, Jilin, Heilongjiang, and Shanghai
	**Low PTE**	**High PTE**

## Conclusions and suggestions

This study employed a three-stage DEA model to analyze the efficiency of SSE in 31 provinces and municipalities in China from 2007 to 2016. The conclusions are as follows:

1. The overall efficiency of SSE in China is not high. The results of stage 3 showed that the efficiency level of 29 provinces/municipalities did not reach the efficiency frontier, indicating that further general improvement is needed. However, such efficiency did increase throughout the research period, indicating that the overall efficiency is improving.
2. Environmental factors and statistical noises have a significant impact on the efficiency of SSE in China. In addition, the influence of environmental factors appears to be more dominant. Specifically, per capita GDP and marketization level are not conducive to the improvement of efficiency, while urbanization level and financial autonomy are conducive to the promotion of efficiency. After eliminating the influences of environmental factors, TE, PTE, and SE of all regions increased. The increase in TE was due to the improvement in both PE and SE. These findings suggested that if environmental factors and statistical noises are not considered, the efficiency of SSE in China is likely to be underestimated.
3. The regional differences in the efficiency of SSE were significant and ranked by descending order as follows: central region, eastern region and western region. The SE of the eastern region and PTE of the western region were lower than the national average, which has limited the efficiency levels of the two regions.

Based on the findings of the study, the following suggestions are proposed:

1. Regional governments should implement differentiated strategies to improve their management level and input scale according to the regional conditions. According to the results, provinces/municipalities with high PTE and low SE, such as Beijing and Tianjin, should maintain the current allocation and management level and strive to adjust investment in social security so as to achieve an optimal scale. Provinces/municipalities with low PTE and high SE, such as Shanxi and Inner Mongolia, should maintain the existing financial investment in social security and promote efficiency by improving expenditure management and corresponding management ideologies. Since both Hainan and Qinghai have low PTE and SE, they should increase their investment in social security while improving the allocation and management of funds.
2. The government should be wary to the impact of environmental factors (such as regional economic development, urbanization level, marketization level, and financial autonomy) on the efficiency of SSE. First, it is suggested to promote the construction of new urbanization; actively guide, cooperate, and participate in the free flow of production factors; narrow the gap between urban and rural regions for the provision of public goods and services; and improve infrastructure so as to maximize the promotion effect of urbanization on the social security system in both urban and rural areas. Second, the government should optimize and improve the regional industrial structure and avoid the excessive pursuit of the rapid development of tertiary industries. A balanced development of the three major industries is necessary for stable growth. Third, the government should promote the development of the regional economy and improve the financial autonomy of local governments while improving the allocation and management of social security funds.

## Supporting information

S1 Data The raw data of this paper. (RAR)
